# A Novel Strategy for Selective *O*-Methylation of Glycerol in Subcritical Methanol

**DOI:** 10.3389/fchem.2019.00357

**Published:** 2019-05-21

**Authors:** Sophie Bruniaux, Rajender S. Varma, Christophe Len

**Affiliations:** ^1^Sorbonne Universités, Universite de Technologie de Compiegne, Compiègne, France; ^2^Regional Center of Advanced Technologies and Materials, Palacky University, Olomouc, Czechia; ^3^PSL University, Chimie ParisTech, Paris, France

**Keywords:** glycerol, glycerol *O*-methylation, critical methanol, green chemistry, mechanism

## Abstract

A new regioselective approach has been elaborated for the selective conversion of bio-based glycerol into the monomethyl derivative using sub/supercritical methanol. The reaction was realized in a batch process using three reactive components, namely, glycerol, methanol, and potassium carbonate to selectively produce the 3-methoxypropan-1,2-diol in mild yields; the mechanism of the *O*-methylation has been delineated using labeled methanol and GC-MS experiments.

## Introduction

To compensate for the constantly diminishing fossil-derived resources, the concept of biorefinery and the use of vegetable oils has emerged as a promising alternative to meet future challenges in both public and industrial sectors (Biermann et al., 2000; Behr et al., 2008). Glycerol, as a co-product of industrial oleochemistry, is an outstanding example that has significant potential for conversion into valuable products (Len et al., 1996, 1997, 2018; Rafin et al., 2000; DeSousa et al., 2011; Saggadi et al., 2014a,b, 2015; Galy et al., 2017a,b; Nguyen et al., 2017; Varma and Len, 2018). Among the target chemicals, monoalkyl glyceryl ethers (MAGE) are relative chemical inert, which renders them suitable as solvents, chemicals for inks, polymers, lubricants, and liquid detergents (Sutter et al., 2015). Moreover, MAGEs bearing short *O*-alkyl chains have an impact on the energy sector as exemplified by the good fuel additive properties of 3-methoxypropan-1,2-diol (**2**) (Chang et al., 2012). Nevertheless, the main drawback of the use of MAGE is the basic structure of glycerol, which is a symmetrical polyol encompassing three hydroxy (OH) groups, with two identical primary OH and one secondary OH having a similar pKa. To improve the regio- and stereoselectivity starting from glycerol, protection–deprotection steps using solketal (Vanlaldinpuia and Bez, 2011; Jiang et al., 2013; Jakubowska et al., 2015), as well as activation steps deploying glycidol (Cucciniello et al., 2016; Leal-Duaso et al., 2017; Ricciardi et al., 2017, 2018), were necessary to produce the target glycerol ethers, which limits the interest in view of the cost and sustainability. Starting from glycerol (**1**), the direct one-step synthesis of glycerol ethers has been reported using different conventional methods: the Williamson-type synthesis, catalytic *O*-telomerization, acid catalysis, and reductive alkylation (Sutter et al., 2012), among others. Recently, acid homogeneous and heterogeneous catalysts such as Bi(OTf)_3_ (Liu et al., 2013), Amberlyst-15 (Pariente et al., 2009), and silica-supported sulfonic groups (Gu et al., 2008) have been used, with success, for the synthesis of MAGEs. Among the documented research on direct MAGE synthesis, the use of critical alcohol as a solvent and reagent in the presence of homogeneous bases has not been described. In order to provide a greener protocol for the selective etherification of glycerol, herein, we disclose a novel *O*-methylation of glycerol (**1**) into the corresponding 3-methoxypropan-1,2-diol and (**2**) in the presence of a common and widely available homogeneous base under subcritical methanol.

## Experimental Section

### Materials and Methods

Substrates and solvents were purchased from Acros and all materials were used without further purification. Reactions were monitored by TLC (Kieselgel 60F254 aluminum sheet), with detection carried out through the use of an acidic potassium permanganate solution. Column chromatography was performed on silica gel 40–60 μm, while flash column chromatography was accomplished on an automatic apparatus, using silica gel cartridges. ^1^H and ^13^C NMR spectra were recorded on a 400 MHz/54 mm ultralong hold. Chemical shifts (δ) are quoted in parts per million (ppm) and are referenced to TMS as an internal standard with coupling constants (*J*) being quoted in hertz. Gas chromatography analyses are performed on a PerkinElmer gas chromatography (Autosystem XL GC), using an Altech AT HT column (30.0 m, 0.25 mm i.d., 0.1 μm film thickness), with a detector at 300°C, an injector at 350°C, and a constant flow of nitrogen of 1 mL min^−1^. The column is heated at 50°C for 3 min, and then warmed to 270°C with a temperature gradient of 20°C min^−1^ before being held at that temperature for 5 min.

### Synthesis of 3-methoxypropan-1,2-diol (2)

A batch autoclave reactor (100 mL) was charged with glycerol (**1**, 4.0 g, 43.4 mmol), K_2_CO_3_ (1.0 g, 7.2 mmol) and methanol (70 mL) and was sealed, placed in the heating collar, and heated to 220°C under magnetic stirring with an internal control of the temperature and an auto-generated pressure. After 15 h, the autoclave was cooled down to 40°C, with the volatile chemicals subsequently evaporating. The crude product was purified over a column on silica and eluted with a gradient of cyclohexane/ethyl acetate (100:0–0:100, v/v) to afford the pure ether **2** (36%, 1.66 g, 15.6 mmol). ^1^H NMR (400 MHz; CDCl_3_), δ 4.19 (s, 2H, OH), 3.71 (s, 1H, H_2_), 3.61–3.36 (m, 2H, H_1_), 3.36–3.08 (m, 5H, H_3, 4_) ppm; ^13^C NMR (100 MHz; CDCl_3_), δ (ppm): 73.8 (CH, C_3_), 70.6 (CH_2_, C_2_), 63.6 (CH_2_, C_4_), 58.9 (CH_3_, C_1_) ppm.

## Results and Discussion

The optimization of method for the selective *O*-alkylation of glycerol (**1**) was achieved with the glycerol (**1**) and a commercial homogeneous base in methanol. The reaction occurs in an autoclave with a 100 mL container under magnetic stirring under sub- and super-critical conditions. When methanol is subjected to a temperature and a pressure higher than its critical point, supercritical methanol is obtained. When both: (i) the temperature and/or the pressure are lower than that of the critical point; and (ii) the temperature is higher than that of the boiling point with a pressure higher than 1 bar, subcritical methanol is obtained. Subcritical methanol can be referred to as hot compressed methanol. Heating methanol under pressure deeply modifies some of their physical characteristics: density, viscosity, diffusivity, thermal conductivity, static dielectric constant, and ion dissociation constant. In this process, methanol had a dual role of a solvent and as a reagent. Initial studies were performed for glycerol (**1**, 4.0 g, 43.6 mmol) in the presence of K_2_CO_3_ (1.0 g, 7.2 mmol) in methanol (70 mL) for 15 h by varying the temperature from 180 to 260°C (Table 1) wherein the pressure was auto-generated. The use of supercritical methanol (temperature > 239°C and pressure > 81 bar) was not necessary for the *O*-methylation of glycerol (**1**) since a similar result (39% yield) could be obtained in subcritical methanol at 220°C and 60 bar (Table 1, entry 3). Nevertheless, a lower temperature and pressure did not allow the generation of the target *O*-ether (**2**) in a yield higher than 18% (Table 1, entries 4 and 5). The selective direct *O*-alkylation of glycerol (**1**) under our experimental conditions was possible, with the only product observed being the 3-methoxypropan-1,2-diol (**2**).

**Table 1:** *O*-Methylation of glycerol (**1**) in sub- and supercritical methanol under varying temperatures.

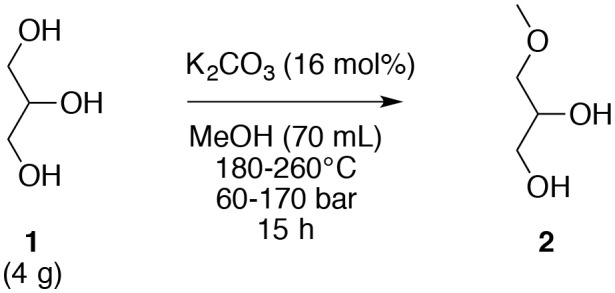			
**Entry**	**Temperature (**°**C)**	**Pressure (bar)**	**Yield of 2 (%)**
1^a^	260	170	39
2^a^	240	160	40
3^b^	220	60	39
4^b^	200	60	18
5^b^	180	50	4

a *Supercritical methanol*.

b *Subcritical methanol*.

Based on these results, different strong and weak bases (16 mol%), including K_2_CO_3_, CaCO_3_, Na_2_CO_3_, Cs_2_CO_3_, (NH_4_)_2_CO_3_, NaOH, KOH, K_3_PO_4_, AcONa, DABCO, Ag_2_O, and Na were evaluated under the initial optimized temperature (220°C). Notably, the strength of the base was not a key factor since strong bases such as NaOH and KOH and weak bases such as AcONa did not furnish the corresponding *O*-methyl derivative **2**. The solubility of the base in subcritical methanol appears to be a rather important parameter. Among the different bases, only K_2_CO_3_ and Cs_2_CO_3_ gave the target MAGE **2** in 39 and 40% yields, respectively. The other carbonate bases did not generate the *O*-ether **2** in satisfactory yields. A plausible explanation may be that the increasing ionic radius among the sodium and the cesium ions could permit a best dissociation of the corresponding salt. Furthermore, this dissociation may be enhanced by the particular properties of the methanol at 220°C and at a 50–70 bar. However, K_2_CO_3_ will be the preferred base in the subsequent evaluations because Cs_2_CO_3_ is more expensive than the corresponding potassium derivative for a rather similar yield (39 vs. 40%). In case of other carbonates explored, the dissociation of the calcium, which is a divalent ion, is harder than the monovalent ion. In case of ammonium counterion, the possibility of the formation of the acid form, and consequently a counter balance of the basic conditions, might be an explanation for the non-formation of 3-methoxypropan-1,2-diol (**2**) with (NH_4_)_2_CO_3_. The use of Na in methanol furnished the corresponding CH_3_ONa base, which gave a poor yield (9%), similar to those obtained with Na_2_CO_3_ and NaOH (Table 2, entry 12).

**Table 2:** Variation of the nature of the base for the *O*-methylation of glycerol (**1**) in subcritical methanol.

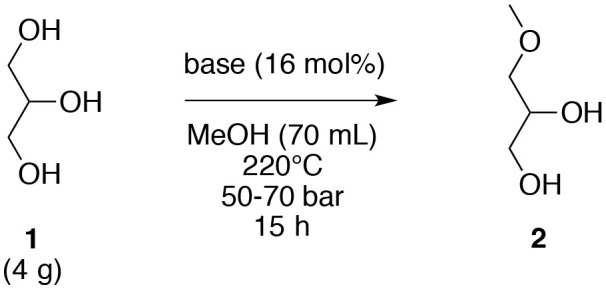		
**Entry**	**Base (16 mol%)**	**Yield of 2 (%)**
1	K_2_CO_3_	39
2	CaCO_3_	0
3	Na_2_CO_3_	8
4	Cs_2_CO_3_	40
5	(NH_4_)_2_CO_3_	0
6	NaOH	4
7	KOH	6
8	K_3_PO_4_	4
9	AcONa	11
10	DABCO	0
11	Ag_2_O	0
12	Na	9

To attain the most efficient process conditions, the highest concentration of glycerol (**1**) in methanol was determined; the variation of glycerol concentration was explored in the presence of K_2_CO_3_ (1.0 g, 7.2 mmol) at 220°C for 15 h. The yield of 3-methoxypropan-1,2-diol (**2**) increased with an increasing glycerol (**1**) concentration to an optimum of 39% yield for a concentration of 57 g L^−1^; a higher concentration of glycerol (**1**, 71 and 86 g L^−1^) produced lower yields of 32 and 29%, respectively. Nevertheless, the *O*-ether productivity was higher when deploying a higher glycerol concentration (5 10^−6^ vs. 4.5 10^−6^ mol s^−1^ L^−1^) (Table 2, entries 4 and 6). Starting with a fixed quantity of glycerol (**1**, 4.0 g), the variation of the amount of K_2_CO_3_ (8.3, 16.6, and 33.4 mol%) showed that 16.6 mol% was the optimum (Table 3, entries 4, 7, and 8); too high an amount of K_2_CO_3_ in methanol may result in the saturation of the media thus impeding the solubility of glycerol (**1**). A scale-down and scale-up study was subsequently undertaken. When a dilution factor of 4 was applied, the reaction was less efficient and only a 19% yield was obtained (Table 3, entry 9), which may be counterbalanced by the increasing base quantity (Table 3, entry 10). On the other hand, when the reaction was scaled up with a factor 2, a similar yield was achieved (36 vs. 39%) (Table 3, entries 4 and 11). Unfortunately, the scale up with a factor 4 resulted in a decreasing yield (Table 3, entries 4 and 12). Furthermore, a test was conducted with the addition of a second lot of K_2_CO_3_ after the first 15 h (Table 3, entry 13). This test gave a better result (44 vs. 39%) but does not justify the additional amount of K_2_CO_3_ and a prolonged reaction time, twice as long.

**Table 3:** Variation of the concentration of glycerol (**1**) and K_2_CO_3_ for the *O*-methylation of glycerol (**1**) in subcritical methanol.

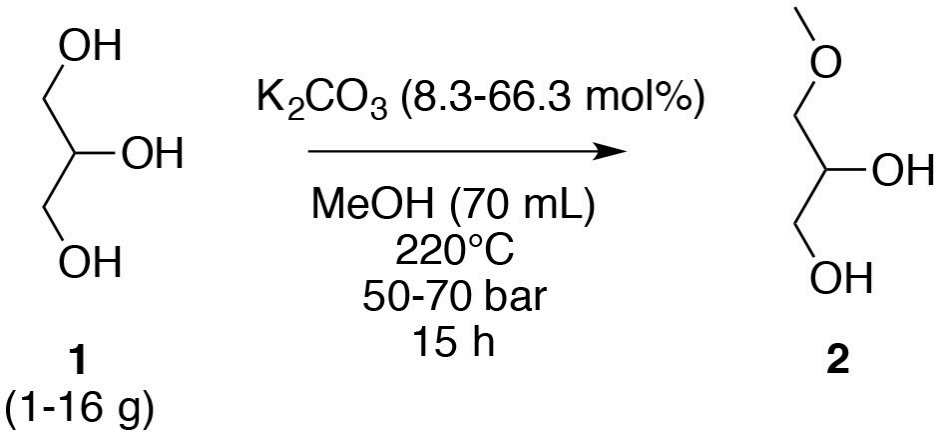			
**Entry**	**Glycerol (g)**	**K**_**2**_**CO**_**3**_ **(mol%)**	**Yield of 2 (%)**
1	1	66.3	11
2	2	33.2	20
3	3	22.10	30
4	4	16.6	39
5	5	13.3	32
6	6	11.0	29
7	4	8.3	33
8	4	33.4	25
9	1	16.5	19
10	1	66.0	37
11	8	16.7	36
12	16	16.6	23
13*	4	2 × 16.6	44

* *Addition of the second amount of K_2_CO_3_ (16.6 mol%) after 15 h for a total reaction time of 30 h*.

Since the present *O*-methylation process is a dehydration-type reaction, the effect of the presence of water was probed; the addition of water (1.4%) in the reaction turned out to be damaging as the yield decreased (Table 4, entries 1 and 3). On the other hand, the use of anhydrous reagents, i.e., glycerol (**1**), K_2_CO_3_ and methanol without water traces was not advantageous because the same results were obtained when the starting materials were not anhydrous (Table 4, entries 1 and 2). This means that the ensuing water during this dehydration was not a critical negative point necessary to make a significant difference. However, a decrease of the reaction yield was noticed when a dryer agent, methyl orthoformiate, was used (Table 4, entry 4), which also means that the water produced may be essential for the reaction.

**Table 4:** Effect of water for the *O*-methylation of glycerol (**1**) in subcritical methanol.

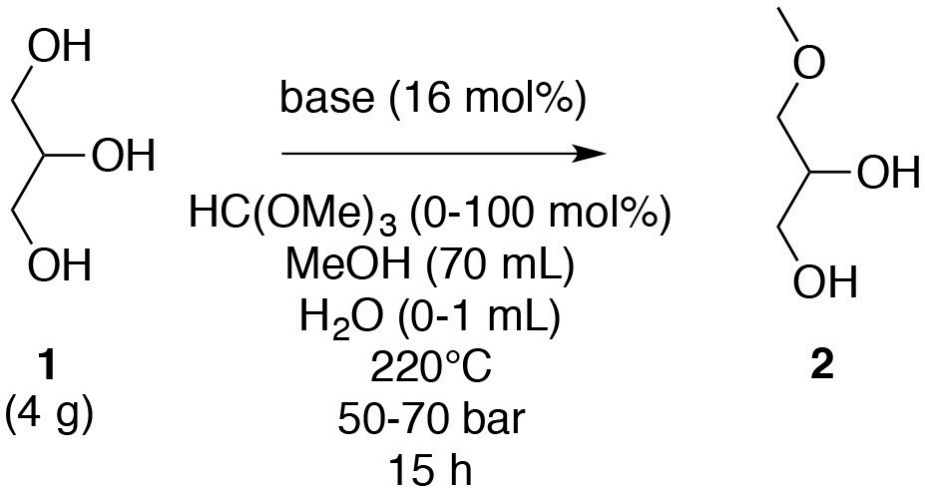			
**Entry**	**Water (mL)**	**HC(OCH**_**3**_**)**_**3**_ **(mol%)**	**Yield of 2 (%)**
1	–	0	39
2	anhydrous	0	35
3	1	0	25
4	–	100	21

**Figure 1:**
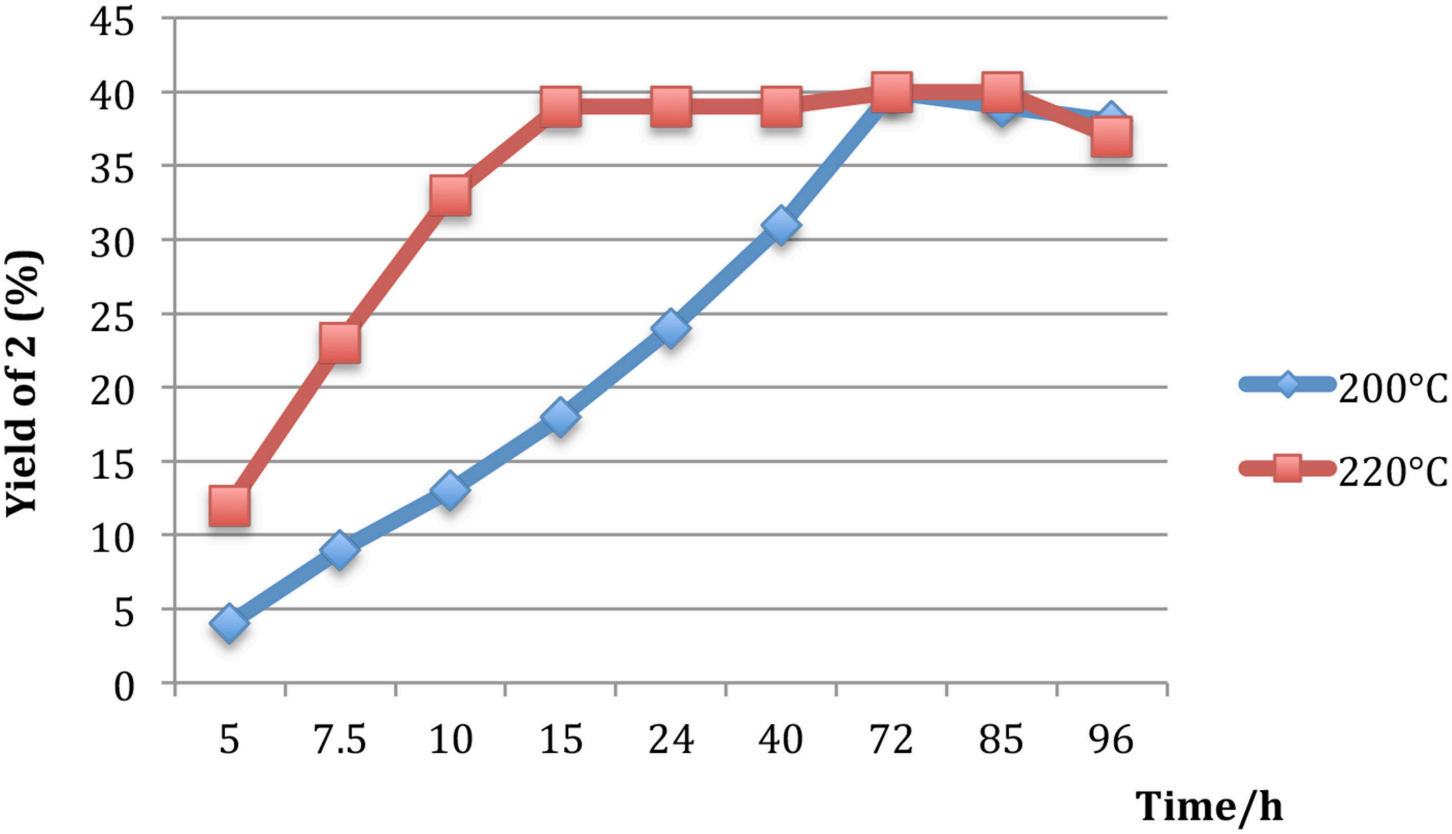
Reaction profile of glycerol conversion to 3-methoxypropan-1,2-diol (**2**) at 200 and 220°C.

The variation of reaction time was next studied at 220 and 200°C; a faster kinetic was observed at 220°C rather than at 200°C. A plateau (40%) was reached with a temperature of 220°C after 15 h and more time (72 h) was required at 200°C to attain a similar yield (Figure 1). In our hands, a longer reaction time was not necessary to acquire a significant increase in the reaction yield.

Based on these results, a scale-down and scale-up study was performed using the following optimized conditions, glycerol (**1**, 4.0 g, 43.6 mmol) in the presence of K_2_CO_3_ (1.0 g, 16.6 mol%) in MeOH (70 mL) at 220°C and at a 50–70 bar for 15 h. Two different reactors (100 and 450 mL) were used to accomplish these evaluations. Indeed, a reactor of 100 mL was used for 20, 50, and 70 mL of methanol and the scale-up with 350 mL was performed in a reactor of 450 mL in size (Table 5). Only a difference of six points (29 vs. 35%) between the smallest volume and the highest volume was discerned, meaning that the results were slightly affected by the scale-down and the scale-up of the reaction.

**Table 5:** Scale down and scale up study for the *O*-methylation of glycerol (**1**) in subcritical methanol.

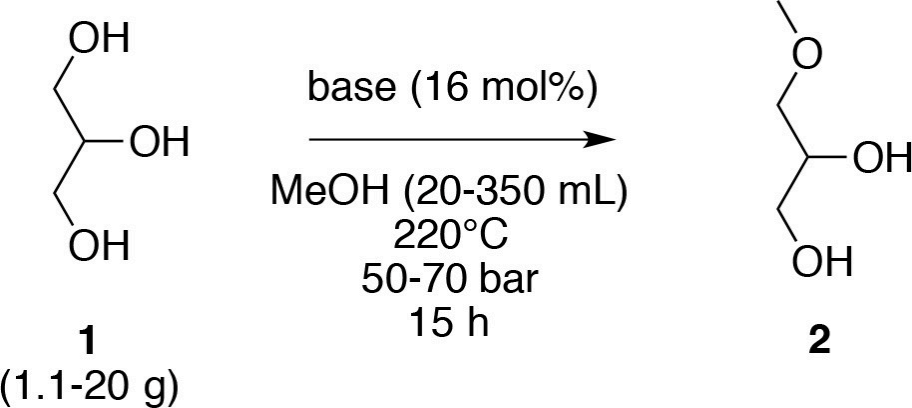		
**Entry**	**Methanol (mL)**	**Yield of 2 (%)**
1	20	29
2	50	30
3	70	39
4	350	35

**Figure 2:**
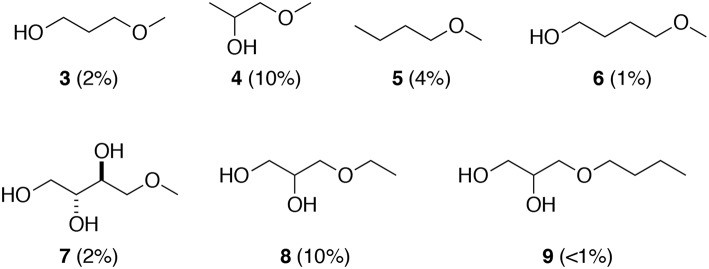
Applications of the *O*-alkylation in subcritical alcohol.

With our optimized reaction conditions in hand, a range of alcohols as substrate and the reagent were screened (Figure 2). Propan-1,3-diol, propan-1,2-diol, erythritol, butan-1,4-diol, and butan-1-ol were evaluated instead of glycerol (**1**) and ethanol and butan-1-ol were evaluated instead of methanol. Changing glycerol (**1**) by another substrate afforded poor yields (< 4%), except for propan-1,2-diol, which furnished the corresponding *O*-methyl ether **4** in a 10% yield; the close proximity of two hydroxyl groups could have had a specific role in the mechanism. However, an unexpected result was obtained with erythritol, which has a structural similarity to glycerol (**1**). The deployment of another solvent other than methanol, notably for ethanol, resulted in a collapse of the yield from 39 to 10%, with only traces of product obtained when butanol was used. Although the low yields were obtained (<10%) in aforementioned cases, only the exclusive regioselective *O*-alkylation of the primary hydroxyl group was observed. One explanation may be the difference of solubility of glycerol (**1**) in the different alcohol, which decreased with the increasing length of the alcohol.

**Figure 3:**
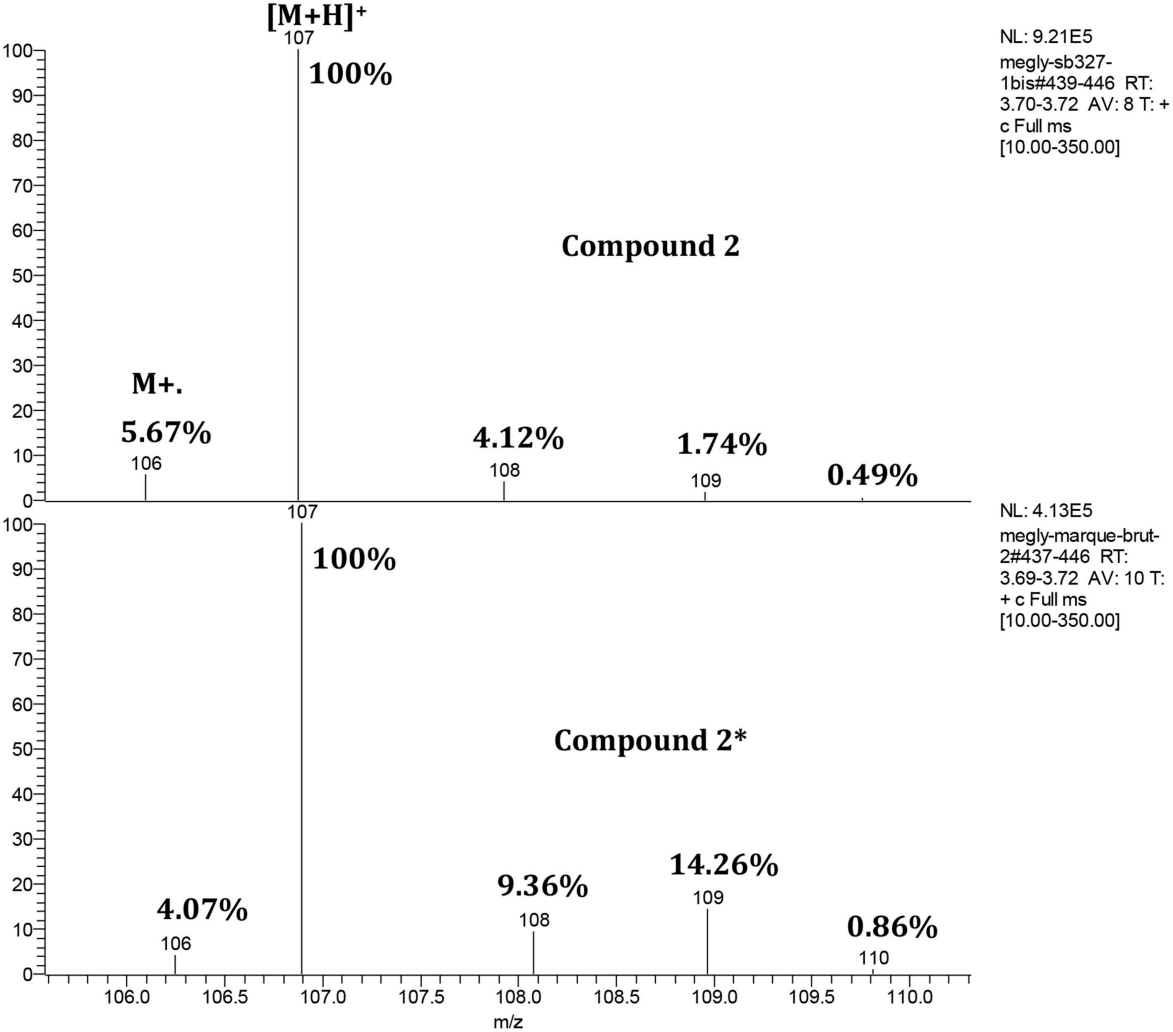
Zoom of the isotopic cluster concerning the GC-MS analysis of compound 2 and compound 2*.

In order to propose a plausible mechanism for this new selective *O*-methylation of glycerol (**1**), the origin of the oxygen atom of the *O*-ether functionality was studied by using methanol labeled with ^18^O. The *O*-alkylation was performed in the presence of glycerol (**1**, 1.14 g) and K_2_CO_3_ (16.6 mol%) in a mixture of methanol MeOH-Me^18^OH (20.4:2.4, v/v) for 15 h at 220°C. After treatment, a GC-MS analysis of the crude target ether 2^*^ was undertaken to identify the oxygen origin of the methoxy group in position 1 of the glycerol moiety (Figure 3). The analysis of the commercial non-labeled 3-methoxypropan-1,2-diol (**2**) showed a signal of the [M+1] which was the corresponding ion with an adding H+ at m/z of 107. An apparition of a signal corresponding to the [M+2] was noticed for the analysis of the crude target ether 2^*^ that matched with the presence of the labeled 3-methoxypropan-1,2-diol (2^*^). To directly access the labeled percent of ^18^O of the 3-methoxypropan-1,2-diol (2^*^), the study was based on the M+2 of the [M+H]^+^. In our case, 12.5% (14.26–1.74%) of labeled ether 2^*^ was obtained, corresponding almost to the introduced Me^18^OH for the *O*-alkylation. Consequently, the oxygen atom of the ether function came from the methanol, which was used as a solvent and a reagent.

**Scheme 1:**
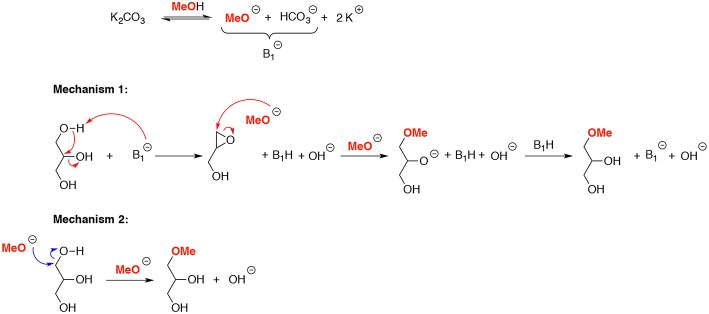
Plausible mechanism for *O*-methylation of glycerol in sucritical methanol.

Two possible reaction mechanisms may be suggested based under these basic conditions: (i) one with the direct regioselective nucleophile attack of the methoxide to the primary hydroxyl group of glycerol (**1**); (ii) one with the regioselective epoxide ring opening (Scheme 1). In this case, the glycerol epoxide was an intermediate obtained by successive formation of the glycerolate, followed by an internal nucleophilic addition of the oxygen atom to the carbon atom at position 2 to form the corresponding epoxide.

## Conclusions

A simple batch protocol has been developed for the regioselective *O*-methylation of glycerol in the presence of an inexpensive commercial base K_2_CO_3_ in subcritical methanol. Moreover, a scale-up of the process can be achieved using autoclaves without a loss in neither reactivity nor selectivity; a 39% yield was obtained starting from glycerol (**1**, 4.0 g) vs. a 35% yield from the higher amount of glycerol (**1**, 20.0 g). Conventional flash chromatography was carried out with success to obtain the pure target methyl ether without further purification. A mechanistic pathway has been delineated to determinate the origin of the oxygen of the methoxy group, which came from the methanol following the results of the labeled reaction. To date, the additional tests to extend the method to other substrates have not provided significant results (<10%). A more in-depth study is warranted to enhance the scope of the proposed environmentally friendly *O*-alkylation of glycerol to different alcohols in batch reactors as well as in continuous flow operations.

## Author Contributions

SB, RV, and CL analyzed the bibliography. SB performed the experiment. RV and CL wrote the paper.

### Conflict of Interest Statement

The authors declare that the research was conducted in the absence of any commercial or financial relationships that could be construed as a potential conflict of interest.
